# Supplementary material for: Impact of the Liberian National Community Health Assistant Program on childhood illness care in Grand Bassa County, Liberia
Source: PLOS Glob Public Health. 2022 Jun 30;2(6):e0000668. doi: 10.1371/journal.pgph.0000668 (PMC10021826; doi:10.1371/journal.pgph.0000668)
Supplement: S2 Appendix — (DOCX) [file pgph.0000668.s002.docx]

**Supplement 3. Sensitivity analyses**

Regression adjustment: This analysis replicates the main analysis except that confounders are adjusted by regression rather than inverse probability of treatment weighting.

Table 1. Difference-in-Differences in Childhood Illness Care

|  | Sample size  (unweighted) | | Unadjusted Model | | Regression-Adjusted Model | |
| --- | --- | --- | --- | --- | --- | --- |
| Care-seeking from qualified provider | Pre | Post | DID %  (95% CI) | P | DID %  (95% CI) | P |
| Any illness | 894 | 397 | 55.2  (41.2, 69.3) | <0.001 | 56.8  (42.9, 70.7) | <0.001 |
| Fever | 691 | 293 | 60.7  (45.1, 76.2) | <0.001 | 64.5  (49.6, 79.3) | <0.001 |
| Diarrhea | 590 | 215 | 69.8  (54.5, 85.1) | <0.001 | 67.2  (52.2, 82.2) | <0.001 |
| Acute Respiratory Illness | 295 | 79 | 39.1  (10.9, 67.2) | 0.007 | 38.5  (13.6, 63.4) | 0.003 |
| Oral rehydration therapy for diarrhea | 586 | 215 | 32.9  (15.0, 50.7) | <0.001 | 32.1  (14.8, 49.4) | <0.001 |
| Rapid diagnostic test for fever | 686 | 293 | 41.9  (26.0, 57.8) | <0.001 | 43.5  (28.3, 58.6) | <0.001 |

Restricted only to agricultural areas: This analysis replicates the main analysis except that it excludes respondents who live in mining communities. The main analysis could not adjust for community type by IPTW because too few respondents were from mining communities.

Table 2. Difference-in-Differences in Childhood Illness Care

|  | Sample size  (unweighted) | | Unadjusted Model | | Inverse Probability of Treatment Weighted Model | |
| --- | --- | --- | --- | --- | --- | --- |
| Care-seeking from qualified provider | Pre | Post | DID %  (95% CI) | P | DID %  (95% CI) | P |
| Any illness | 825 | 360 | 53.2  (38.7, 67.7) | <0.001 | 61.0  (45.2, 76.9) | <0.001 |
| Fever | 636 | 264 | 58.5  (42.2, 74.8) | <0.001 | 64.9  (47.5, 82.3) | <0.001 |
| Diarrhea | 547 | 199 | 69.5  (54.7, 84.4) | <0.001 | 75.5  (60.0, 90.9) | <0.001 |
| Acute Respiratory Illness | 275 | 73 | 37.6  (7.9, 67.2) | 0.013 | 50.1  (18.3, 81.9) | 0.002 |
| Oral rehydration therapy for diarrhea | 543 | 199 | 30.7  (12.4, 48.9) | 0.001 | 37.5  (19.0, 56.1) | <0.001 |
| Rapid diagnostic test for fever | 631 | 264 | 39.4  (22.6, 56.1) | <0.001 | 38.2  (18.8, 57.5) | <0.001 |

Does not exclude one enumerator’s data: In the main analysis, we excluded data from one enumerator because of data quality concerns. This analysis replicates the main analysis except that it includes that enumerator’s data.

Table 3. Difference-in-Differences in Childhood Illness Care

|  | Sample size  (unweighted) | | Unadjusted Model | | Inverse Probability of Treatment Weighted Model | |
| --- | --- | --- | --- | --- | --- | --- |
| Care-seeking from qualified provider | Pre | Post | DID %  (95% CI) | P | DID %  (95% CI) | P |
| Any illness | 894 | 463 | 55.4  (42.5, 68.2) | <0.001 | 59.4  (45.6, 73.2) | <0.001 |
| Fever | 691 | 351 | 60.4  (46.1, 74.7) | <0.001 | 64.7  (50.1, 79.3) | <0.001 |
| Diarrhea | 590 | 277 | 65.4  (51.4, 79.4) | <0.001 | 68.0  (52.5, 83.4) | <0.001 |
| Acute Respiratory Illness | 295 | 127 | 50.4  (28.5, 72.2) | <0.001 | 55.4  (32.5, 78.4) | <0.001 |
| Oral rehydration therapy for diarrhea | 586 | 277 | 40.3  (24.8, 55.8) | <0.001 | 39.5  (23.4, 55.6) | <0.001 |
| Rapid diagnostic test for fever | 686 | 350 | 39.8  (24.1, 55.5) | <0.001 | 37.2  (19.4, 55.0) | <0.001 |
