# Supplementary material for: Impact of the Liberian National Community Health Assistant Program on childhood illness care in Grand Bassa County, Liberia
Source: PLOS Glob Public Health. 2022 Jun 30;2(6):e0000668. doi: 10.1371/journal.pgph.0000668 (PMC10021826; doi:10.1371/journal.pgph.0000668)
Supplement: S1 Code — (RTF) [file pgph.0000668.s004.rtf]

/*Notes1. Most commands are standard in Stata 17. However, the following addtional commands may need to be installed:	- sencode	- grc1leg	- Additionally, manuscript plots use the plotplainblind and plotplain scheme.	2. Because the journal does not accept .do files as supplements, this file will need to be copied into a do file.3. Replication data are available at https://data.mendeley.com/datasets/7h9f9mkh9b/1.*/use lms_gb_2019_child_did.dta, clearsvyset clusterid [pweight=weight_sampling_use], strata(strata) fpc(fpc1_use) vce(linearized) singleunit(centered) || id_household, fpc(fpc2_use) *=======================================================================================*=======================================================================================* Table 1*=======================================================================================*=======================================================================================* Table 1 uses only survey weights and FPCssvyset clusterid [pweight=weight_sampling_use], strata(strata) fpc(fpc1_use) vce(linearized) singleunit(centered) || id_household, fpc(fpc2_use) 	foreach var of varlist mining_community gender_child_health mother_educ_cat femalelanguage married illness_gt1 {	svy, subpop(if illness==1): tab `var' did_level, col ci format(%9.3f)	}foreach var of varlist age_child_months health_fac_dist_use num_child_u1 num_child_u5 age {	svy, subpop(if illness==1): mean `var', over(did_level) cformat(%9.1f)}			svy, subpop(if illness==1): mean gb_wealth_index_2018, over(did_level) cformat(%9.2f)						*=======================================================================================*=======================================================================================* Table 2*=======================================================================================*=======================================================================================*=======================================================================================* IPTW*=======================================================================================/* Covariates:  household wealth quintile (calculated via principal component analysis from an asset index via the Filmer–Pritchett method), quintile of distance to nearest health facility, mother's formal education (none, some primary, completed primary or higher,child's age (months), mother's age (years), mother's primary language (English or Bassa), number of illnesses per child in past 2 weeks (1 vs ≥),  number of children under 5 in the household, child gender*/* Look at mining community with other variables to understand why it breaks the IPTW modelsvy, subpop(if illness == 1): tab mining_community did_level, col ci // no mining communities in pre-treatment groupsvy, subpop(if illness == 1): tab mining_community gb_wealth_qtile, col cisvy, subpop(if illness == 1): tab mining_community distancetoclinic_qtile, col cisvy, subpop(if illness == 1): tab mining_community mother_educ_cat, col cisvy, subpop(if illness == 1): tab mining_community femalelanguage, col cisvy, subpop(if illness == 1): tab mining_community illness_gt1, col cisvy, subpop(if illness == 1): tab mining_community num_child_u5, col cigen ln_distance = ln(health_fac_dist_use)	label var ln_distance "log of distance to health facility"		** Used a log-transformed distance variable, which should better stabilize its variance. // Generate IPT weights and run balance diagnostics.	set seed 12345	gen outcome_random=uniform() if illness==1 & treatment_formal!=.	// Generates a random variable for balance diagnostics. Sets all obs w/o illness or with missing treatment data to missing. Results in the same number of observations as in the diff-in-diff analyses.				gen outcome_tx_rand=0 if outcome_random<=.5	replace outcome_tx_rand=1 if outcome_random>.5 & outcome_random<.	label var outcome_tx_rand "Random care seeking variable: USE FOR BALANCE DIAGNOSTICS ONLY!"		teffects ipw (outcome_tx_rand) (did_level i.gender_child_health i.gb_wealth_qtile c.ln_distance i.mother_educ_cat c.age_child_months c.age i.femalelanguage i.illness_gt1 c.num_child_u5) if illness==1 & outcome_tx_rand!=. [pw=weight_sampling_use] , pomeans 		tebalance summarize		matrix balance_dx = r(table)			predict pscore_tx1 pscore_tx2 pscore_tx3 pscore_tx4 if e(sample)==1, ps 	gen ipw_tx_ate=1/pscore_tx1 if did_level==1	replace ipw_tx_ate=1/pscore_tx2 if did_level==2	replace ipw_tx_ate=1/pscore_tx3 if did_level==3 	replace ipw_tx_ate=1/pscore_tx4 if did_level==4	label var ipw_tx_ate "IPTW for childhood treatment analysis"	* Multiply inverse of p-score to sampling weight to get the correct IPT weights		gen iptw_weight = ipw_tx_ate*weight_sampling_use if ipw_tx_ate != .		label var iptw_weight "IPT weight: use for main analyses"				drop ipw_tx_ate		putexcel set balance_output, replace		putexcel A1=("group") B1=("covariate") C1=("raw_sd") D1=("weighted_sd") E1=("raw_var_rat") F1=("weighted_var_rat")		putexcel A2=matrix(balance_dx), rownames					preserve		import excel balance_output, first clear			replace raw_sd = abs(raw_sd)			replace weighted_sd = abs(weighted_sd)						gen group_covariate = group + " " + covariate			 			sencode group_covariate, generate(cov_code) gsort(raw_sd)						label define cov_code2 1 "maternal age (comparison, post)" ///			2 "3rd wealth quintile (intervention, post)" ///			3 "gender (comparison, post)" ///			4 "gender (comparison, pre)" ///			5 "maternal age (intervention, post)" ///			6 "4th wealth quintile (intervention, post)" ///			7 "maternal education (comparison, pre)" ///			8 "log of distance (intervention, post)" ///			9 "child age (intervention, post)" /// 			10 "language (comparison, pre)" ///			11 "3rd wealth quintile (comparison, pre)" ///			12 "4th wealth quintile (comparison, pre)" ///			13 "2nd wealth quintile (comparison, post)" ///			14 "number of children (comparison, pre)" ///			15 "maternal age (comparison, pre)" ///			16 "highest wealth quintile (comparison, pre)" ///			17 "maternal education (intervention, post)" ///			18 "child age (comparison, post)" ///			19 "highest wealth quintile (comparison, post)" ///			20 "maternal education (intervention, post)" ///			21 "number of illnesses (comparison, pre)" /// 			22 "2nd wealth quintile (intervention, post)" /// 			23 "gender (intervention, post)" ///			24 "number of children (intervention, post)" ///			25 "4th wealth quintile (comparison, post)" ///			26 "maternal education (comparison, post)" ///			27 "3rd wealth quintile (comparison, post)" ///			28 "2nd wealth quintile (comparison, pre)" ///			29 "maternal education (comparison, pre)" ///			30 "maternal education (comparison, post)" ///			31 "child age (comparison, pre)" ///			32 "log of distance (comparison, pre)" ///			33 "language (comparison, post)" ///			34 "highest wealth quintile (intervention, post)" ///			35 "number of children (comparison, post)" ///			36 "log of distance (comparison, post)" ///			37 "number of illnesses (intervention, post)" ///			38 "language (intervention, post)" ///			39 "number of illnesses (comparison, post)" 						label val cov_code cov_code2									describe			local n = r(N)						twoway scatter cov_code raw_sd, name(st_diff, replace) 				graph addplot scatter cov_code weighted_sd, ylabel(1(1)`n', labsize(vsmall) angle(horizontal) valuelabel labcolor(gs3)) xline(.1, lcolor(gray) lpattern(-..)) xline(.25, lcolor(gray) lpattern(_)) ///					graphregion(margin(l+5)) title(Covariate Balance Before and After IPTW, margin(l-25)) ytitle("") xtitle("standardized differences") legend(order(2 "weighted" 1 "unweighted")) 			 			restore											// MAKE SURE USING THE CORRECT WEIGHTS! MAKE SURE HAVE RE-SVYSET FROM OTHER ANALYSES.	svyset clusterid [pweight=iptw_weight], strata(strata) fpc(fpc1_use) vce(linearized) singleunit(centered) || id_household, fpc(fpc2_use) 			*NOTE: Single sampling unit for one of the 2018 strata - set to centered to proceed with analysis		* All illnesses*Ntab survey if illness == 1svy, subpop(if illness == 1): regress treatment_formal i.lmh_managed##i.survey 	local did = round(r(table)[1,8] * 100, .1)	di `did'margins i.survey#i.lmh_managed, subpop(if illness==1) vce(uncond)marginsplot, plot1opts(lwidth(medthick) lpattern(dash) lcolor(gs8) mcolor(gs8)) ci1opts(lcolor(gs8)) plot2opts(lpattern(solid) lcolor(black) lwidth(medthick) mcolor(black)) ci2opts(lcolor(black)) title("Care by a Qualified Provider, All Illnesses") ytitle("Proportion Receiving Care from Qualified Provider", size(vsmall)) xtitle("", size(small)) xlabel(1 "pre" 2 "post")  legend(order(2 "intervention" 1 "comparison") rows(1)) yscale(range(0 1)) ylabel(0(0.2)1) text(.75 1.5 "DiD=`did'", size(small) just(right)) name(composite, replace)margins r.survey, over(lmh_managed) subpop(if illness==1) vce(unconditional)margins r.lmh_managed#r.survey, subpop(if illness==1) vce(unconditional)* Fever* ntab survey if fever_last2wks == 1svy, subpop(if fever_last2wks == 1): regress treatment_formal i.lmh_managed##i.survey 	local did = round(r(table)[1,8] * 100, .1)	di `did'margins i.survey#i.lmh_managed, subpop(if fever_last2wks==1) vce(uncond)marginsplot, plot1opts(lwidth(medthick) lpattern(dash) lcolor(gs8) mcolor(gs8)) ci1opts(lcolor(gs8)) plot2opts(lpattern(solid) lcolor(black) lwidth(medthick) mcolor(black)) ci2opts(lcolor(black)) title("Fever Care by a Qualified Provider") ytitle("Proportion Receiving Care from Qualified Provider", size(vsmall)) xtitle("", size(small)) xlabel(1 "pre" 2 "post") legend(order(2 "intervention" 1 "comparison")) yscale(range(0 1)) ylabel(0(0.2)1) text(.75 1.5 "DiD=`did'", size(small) just(right)) name(fever, replace)margins r.survey, over(lmh_managed) subpop(if fever_last2wks==1) vce(unconditional)margins r.lmh_managed#r.survey, subpop(if fever_last2wks==1) vce(unconditional)* Diarrhea* ntab survey if diarrhea_last2wks == 1svy, subpop(if diarrhea_last2wks == 1): regress treatment_formal i.lmh_managed##i.survey 	local did = round(r(table)[1,8] * 100, .1)	di `did'margins i.survey#i.lmh_managed, subpop(if diarrhea_last2wks==1) vce(uncond)marginsplot, plot1opts(lwidth(medthick) lpattern(dash) lcolor(gs8) mcolor(gs8)) ci1opts(lcolor(gs8)) plot2opts(lpattern(solid) lcolor(black) lwidth(medthick) mcolor(black)) ci2opts(lcolor(black)) title("Diarrhea Care by a Qualified Provider") ytitle("Proportion Receiving Care from Qualified Provider", size(vsmall)) xtitle("", size(small)) xlabel(1 "pre" 2 "post") legend(order(2 "intervention" 1 "comparison")) yscale(range(0 1)) ylabel(0(0.2)1) text(.75 1.5 "DiD=`did'", size(small) just(right)) name(diarrhea, replace)margins r.survey, over(lmh_managed) subpop(if diarrhea_last2wks==1) vce(unconditional)margins r.lmh_managed#r.survey, subpop(if diarrhea_last2wks==1) vce(unconditional)* ARI* ntab survey if ari_last2wks == 1svy, subpop(if ari_last2wks == 1): regress treatment_formal i.lmh_managed##i.survey 	local did = round(r(table)[1,8] * 100, .1)	di `did'margins i.survey#i.lmh_managed, subpop(if ari_last2wks==1) vce(uncond)marginsplot, plot1opts(lwidth(medthick) lpattern(dash) lcolor(gs8) mcolor(gs8)) ci1opts(lcolor(gs8)) plot2opts(lpattern(solid) lcolor(black) lwidth(medthick) mcolor(black)) ci2opts(lcolor(black)) title("ARI Care by a Qualified Provider") ytitle("Proportion Receiving Care from Qualified Provider", size(vsmall)) xtitle("", size(small)) xlabel(1 "pre" 2 "post") legend(order(2 "intervention" 1 "comparison")) yscale(range(0 1)) ylabel(0(0.2)1) text(.75 1.5 "DiD=`did'", size(small) just(right)) name(ari, replace)margins r.survey, over(lmh_managed) subpop(if ari_last2wks==1) vce(unconditional)margins r.lmh_managed#r.survey, subpop(if ari_last2wks==1) vce(unconditional)* ORT for diarrhea* ntab diarrhea_rcvd_ors survey if diarrhea_last2wks == 1svy, subpop(if diarrhea_last2wks == 1): regress diarrhea_rcvd_ors i.lmh_managed##i.survey 	local did = round(r(table)[1,8] * 100, .1)	di `did'margins i.survey#i.lmh_managed, subpop(if diarrhea_last2wks==1) vce(uncond)marginsplot, plot1opts(lwidth(medthick) lpattern(dash) lcolor(gs8) mcolor(gs8)) ci1opts(lcolor(gs8)) plot2opts(lpattern(solid) lcolor(black) lwidth(medthick) mcolor(black)) ci2opts(lcolor(black)) title("Received Oral Rehydrative Therapy for Diarrhea") ytitle("Proportion Receiving ORT", size(vsmall)) xtitle("", size(small)) xlabel(1 "pre" 2 "post") legend(order(2 "intervention" 1 "comparison")) yscale(range(0 1)) ylabel(0(0.2)1) text(.75 1.5 "DiD=`did'", size(small) just(right)) name(ors, replace)margins r.survey, over(lmh_managed) subpop(if diarrhea_last2wks==1) vce(unconditional)margins r.lmh_managed#r.survey, subpop(if diarrhea_last2wks==1) vce(unconditional)* RDT for fever* ntab fever_rcvd_rdt survey if fever_last2wks == 1svy, subpop(if fever_last2wks == 1): regress fever_rcvd_rdt i.lmh_managed##i.survey 	local did = round(r(table)[1,8] * 100, .1)	di `did'margins i.survey#i.lmh_managed, subpop(if fever_last2wks==1) vce(uncond)marginsplot, plot1opts(lwidth(medthick) lpattern(dash) lcolor(gs8) mcolor(gs8)) ci1opts(lcolor(gs8)) plot2opts(lpattern(solid) lcolor(black) lwidth(medthick) mcolor(black)) ci2opts(lcolor(black)) title("Received Malaria Rapid Diagnostic Test if Had Fever") ytitle("Proportion Receiving RDT", size(vsmall)) xtitle("", size(small)) xlabel(1 "pre" 2 "post") legend(order(2 "intervention" 1 "comparison")) yscale(range(0 1)) ylabel(0(0.2)1) text(.75 1.5 "DiD=`did'", size(small) just(right)) name(rdt, replace)margins r.survey, over(lmh_managed) subpop(if fever_last2wks==1) vce(unconditional)margins r.lmh_managed#r.survey, subpop(if fever_last2wks==1) vce(unconditional)grc1leg composite ari diarrhea ors fever rdt, r(3) legendfrom(composite) title(Childhood Illness Care Difference-in-Differences) name(combined, replace)	graph display combined, xsize(6) ysize(8)  		*=======================================================================================* Unadjusted regression model*=======================================================================================* Run SVYSET command to apply survey design weights and finite population correctionsvyset clusterid [pweight=weight_sampling_use], strata(strata) fpc(fpc1_use) vce(linearized) singleunit(centered) || id_household, fpc(fpc2_use) * Compositesvy, subpop(if illness == 1): regress treatment_formal i.lmh_managed##i.survey margins r.lmh_managed#r.survey, subpop(if illness==1) vce(unconditional)* Feversvy, subpop(if fever_last2wks == 1): regress treatment_formal i.lmh_managed##i.survey margins r.lmh_managed#r.survey, subpop(if fever_last2wks==1) vce(unconditional)* Diarrheasvy, subpop(if diarrhea_last2wks == 1): regress treatment_formal i.lmh_managed##i.survey margins r.lmh_managed#r.survey, subpop(if diarrhea_last2wks==1) vce(unconditional)* ARIsvy, subpop(if ari_last2wks == 1): regress treatment_formal i.lmh_managed##i.survey margins r.lmh_managed#r.survey, subpop(if ari_last2wks==1) vce(unconditional)* ORT for diarrheasvy, subpop(if diarrhea_last2wks == 1): regress diarrhea_rcvd_ors i.lmh_managed##i.survey margins r.lmh_managed#r.survey, subpop(if diarrhea_last2wks==1) vce(unconditional)* RDT for feversvy, subpop(if fever_last2wks == 1): regress fever_rcvd_rdt i.lmh_managed##i.survey margins r.lmh_managed#r.survey, subpop(if fever_last2wks==1) vce(unconditional)	*=======================================================================================*=======================================================================================* Figure - Providers seen for child illness*=======================================================================================*=======================================================================================// MAKE SURE USING THE CORRECT WEIGHTS! MAKE SURE HAVE RE-SVYSET FROM OTHER ANALYSES.svyset clusterid [pweight=iptw_weight], strata(strata) fpc(fpc1_use) vce(linearized) singleunit(centered) || id_household, fpc(fpc2_use) 			local providers prov_chw prov_hospital prov_gchv prov_blackbagger prov_drugstore prov_tradsvy, subpop(if illness==1 & survey==1 & lmh_managed==1): mean `providers'	matrix providers_pre_int = e(b)*100svy, subpop(if illness==1 & survey==1 & lmh_managed==0): mean `providers'		matrix providers_pre_comp = e(b)*100		svy, subpop(if illness==1 & survey==2 & lmh_managed==1): mean `providers'	matrix providers_post_int = e(b)*100	svy, subpop(if illness==1 & survey==2 & lmh_managed==0): mean `providers'	matrix providers_post_comp = e(b)*100	putexcel set providers_output, replace		putexcel A1=("chw") B1=("hospital") C1=("gchv") D1=("blackbagger") E1=("drugstore") F1=("traditional") G1=("intervention") H1=("time")		putexcel A2=matrix(providers_pre_int)		putexcel G2=("intervention")		putexcel H2=("pre")		putexcel A3=matrix(providers_pre_comp)		putexcel G3=("comparison")		putexcel H3=("pre")			putexcel A4=matrix(providers_post_int)		putexcel G4=("intervention")		putexcel H4=("post")		putexcel A5=matrix(providers_post_comp)		putexcel G5=("comparison")		putexcel H5=("post")			putexcel save	preserve		import excel providers_output, first clear		graph bar chw hospital gchv blackbagger drugstore traditional if time=="pre" & intervention=="intervention", bar(1, color(black%100)) bar(2, color(black%85)) bar(3, color(black%70)) bar(4, color(black%45)) bar(5, color(black%30)) bar(6, color(black%15)) ytitle(percent) title(Before Implementation, size(med)) blabel(bar, format(%9.1f)) ylabel(0(20)100) legend(order(1 "CHA" 2 "hospital" 3 "GCHV" 4 "blackbagger" 5 "drugstore" 6 "traditional") rows(1)) name(prov_pre_int, replace) 		graph bar chw hospital gchv blackbagger drugstore traditional if time=="post" & intervention=="intervention", bar(1, color(black%100)) bar(2, color(black%85)) bar(3, color(black%70)) bar(4, color(black%45)) bar(5, color(black%30)) bar(6, color(black%15)) ytitle(percent) title(After Implementation, size(med)) blabel(bar, format(%9.1f)) ylabel(0(20)100) name(prov_post_int, replace)		graph bar chw hospital gchv blackbagger drugstore traditional if time=="pre" & intervention=="comparison",  bar(1, color(black%100)) bar(2, color(black%85)) bar(3, color(black%70)) bar(4, color(black%45)) bar(5, color(black%30)) bar(6, color(black%15)) ytitle(percent) title(Before Implementation, size(med)) blabel(bar, format(%9.1f)) ylabel(0(20)100) name(prov_pre_comp, replace)		graph bar chw hospital gchv blackbagger drugstore traditional if time=="post" & intervention=="comparison",  bar(1, color(black%100)) bar(2, color(black%85)) bar(3, color(black%70)) bar(4, color(black%45)) bar(5, color(black%30)) bar(6, color(black%15)) ytitle(percent) title(After Implementation, size(med)) blabel(bar, format(%9.1f)) ylabel(0(20)100) name(prov_post_comp, replace)		*grc1leg prov_pre_int prov_post_int prov_pre_comp prov_post_comp, ycommon title(Percent of Children Receiving Treatment from Each Provider Type) name(prov_combined, replace)			grc1leg prov_pre_int prov_post_int, ycommon title(Intervention Areas) name(prov_int_combined, replace)		grc1leg prov_pre_comp prov_post_comp, ycommon title(Comparison Areas) name(prov_comp_combined, replace)		grc1leg prov_int_combined prov_comp_combined, r(2) title(Percent of Sick Children Receiving Care from Each Provider Type) name(prov_combined, replace)			restore						*=======================================================================================*=======================================================================================* EQUITY ANALYSIS*=======================================================================================*=======================================================================================	// MAKE SURE USING THE CORRECT WEIGHTS! MAKE SURE HAVE RE-SVYSET FROM OTHER ANALYSES.svyset clusterid [pweight=weight_sampling_use], strata(strata) fpc(fpc1_use) vce(linearized) singleunit(centered) || id_household, fpc(fpc2_use) 			* Recategorize distance to nearest facility - <10 km or >10 kmgen distancetoclinic_u10 = 0 if health_fac_dist_use >= 10 & health_fac_dist_use < .replace distancetoclinic_u10 = 1 if health_fac_dist_use < 10 label var distancetoclinic_u10 "Distance to nearest health facility"label define distance10 1 "5-<10 km" 0 "10+ km", replacelabel value distancetoclinic_u10 distance10tab distancetoclinic_u10 , miss*** Distance equity ****svy, subpop(if illness == 1 & survey==1): prop treatment_formal, over(distancetoclinic_u10)	coeflegend		// difference at baseline	lincom _b[1.treatment_formal@1.distancetoclinic_u10] -  _b[1.treatment_formal@0bn.distancetoclinic_u10]	svy, subpop(if illness == 1): regress treatment_formal i.distancetoclinic_u10##i.lmh_managed##i.survey margins r.lmh_managed#r.survey@i.distancetoclinic_u10, subpop(if illness == 1 ) vce(unconditional)margins r.lmh_managed#r.survey#r.distancetoclinic_u10, subpop(if illness == 1) vce(uncond)svy, subpop(if illness == 1 & lmh_managed==1): logistic treatment_formal i.distancetoclinic_u10#i.survey 	margins i.survey#i.distancetoclinic_u10, subpop(if illness==1 & lmh_managed==1) vce(uncond)		margins r.survey@i.distancetoclinic_u10, subpop(if illness==1 & lmh_managed==1) vce(uncond)		margins r.distancetoclinic_u10@i.survey, subpop(if illness==1 & lmh_managed==1) vce(uncond)		margins r.survey#r.distancetoclinic_u10, subpop(if illness==1 & lmh_managed==1) vce(uncond)	margins i.survey#i.distancetoclinic_u10, subpop(if illness==1 & lmh_managed==1) vce(uncond) predict(xb) saving(distance_margins, replace)						preserve		use distance_margins, clear 			gen estimate = invlogit(_margin)			gen lb = invlogit(_ci_lb)			gen ub = invlogit(_ci_ub)						twoway line estimate _m1 if _m2==0, lcolor(black) || rcap lb ub _m1 if _m2==0, lcolor(black) ///			|| line estimate _m1 if _m2==1, lcolor(gs8) lpattern(longdash) || rcap lb ub _m1 if _m2==1, lcolor(gs8) ///			legend(order(3 "5 to 9.9 km" 1 "10+ km" )) xlabel(1 "pre" 2 "post") ylabel(0(.2)1) ///			title(Distance from Health Facility) xtitle("") name(distance_equity, replace)				restore 		*** Education equity ****svy, subpop(if illness == 1 & survey==1): prop treatment_formal, over(mother_educ_any) coeflegend		// difference at baseline	lincom _b[1.treatment_formal@1.mother_educ_any] -  _b[1.treatment_formal@0bn.mother_educ_any]svy, subpop(if illness == 1): regress treatment_formal i.mother_educ_any##i.lmh_managed##i.survey margins r.lmh_managed#r.survey@i.mother_educ_any, subpop(if illness == 1 ) vce(unconditional)margins r.lmh_managed#r.survey#r.mother_educ_any, subpop(if illness == 1) vce(uncond)svy, subpop(if illness == 1 & lmh_managed==1): logistic treatment_formal i.mother_educ_any#i.survey 	margins i.survey#i.mother_educ_any, subpop(if illness==1 & lmh_managed==1) vce(uncond)	margins r.mother_educ_any@i.survey, subpop(if illness==1 & lmh_managed==1) vce(uncond)	margins r.survey@i.mother_educ_any, subpop(if illness==1 & lmh_managed==1) vce(uncond)	margins r.survey#r.mother_educ_any, subpop(if illness==1 & lmh_managed==1) vce(uncond)	margins i.survey#i.mother_educ_any, subpop(if illness==1 & lmh_managed==1) vce(uncond) predict(xb) saving(education_margins, replace)						preserve		use education_margins, clear 			gen estimate = invlogit(_margin)			gen lb = invlogit(_ci_lb)			gen ub = invlogit(_ci_ub)						twoway line estimate _m1 if _m2==0, lcolor(black) || rcap lb ub _m1 if _m2==0, lcolor(black) ///			|| line estimate _m1 if _m2==1, lcolor(gs8) lpattern(longdash) || rcap lb ub _m1 if _m2==1, lcolor(gs8) ///			legend(order(1 "no education" 3 "some education" )) xlabel(1 "pre" 2 "post") ylabel(0(.2)1) ///			title(Maternal Education) xtitle("") name(education_equity, replace)				restore *** wealth equity ****xtile wealth_median = gb_wealth_index_2018, nq(2)svy, subpop(if illness == 1 & survey==1): prop treatment_formal, over(wealth_median) coeflegend	lincom _b[1.treatment_formal@2.wealth_median] -  _b[1.treatment_formal@1bn.wealth_median]svy, subpop(if illness == 1): regress treatment_formal i.wealth_median##i.lmh_managed##i.survey margins r.lmh_managed#r.survey@i.wealth_median, subpop(if illness == 1 ) vce(unconditional)margins r.lmh_managed#r.survey#r.wealth_median, subpop(if illness == 1) vce(uncond)svy, subpop(if illness == 1 & lmh_managed==1): logistic treatment_formal i.wealth_median#i.survey 	margins i.survey#i.wealth_median, subpop(if illness==1 & lmh_managed==1) vce(uncond)	margins r.wealth_median@i.survey, subpop(if illness==1 & lmh_managed==1) vce(uncond)	margins r.survey@i.wealth_median, subpop(if illness==1 & lmh_managed==1) vce(uncond)	margins r.survey#r.wealth_median, subpop(if illness==1 & lmh_managed==1) vce(uncond)	margins i.survey#i.wealth_median, subpop(if illness==1 & lmh_managed==1) vce(uncond) predict(xb) saving(wealth_margins, replace)						preserve		use wealth_margins, clear 			gen estimate = invlogit(_margin)			gen lb = invlogit(_ci_lb)			gen ub = invlogit(_ci_ub)						twoway line estimate _m1 if _m2==1, lcolor(black) || rcap lb ub _m1 if _m2==1, lcolor(black) ///			|| line estimate _m1 if _m2==2, lcolor(gs8) lpattern(longdash) || rcap lb ub _m1 if _m2==2, lcolor(gs8) ///			legend(order(3 "above median" 1 "below median")) xlabel(1 "pre" 2 "post") ylabel(0(.2)1) ///			title(Household Wealth) xtitle("") name(wealth_equity, replace)				restore graph combine distance_equity education_equity wealth_equity, title(Care from a Qualified Provider in Intervention Areas) name(equity_combined, replace) c(1)	graph display equity_combined, ysize(8) xsize(5.5) 		*=======================================================================================*=======================================================================================* Supplement: Balance diagnostic: IPT weighted version of Table 1.*=======================================================================================*=======================================================================================// MAKE SURE USING THE CORRECT WEIGHTS! MAKE SURE HAVE RE-SVYSET FROM OTHER ANALYSES.svyset clusterid [pweight=iptw_weight], strata(strata) fpc(fpc1_use) vce(linearized) singleunit(centered) || id_household, fpc(fpc2_use) 			foreach var of varlist mining_community gender_child_health mother_educ_cat femalelanguage married illness_gt1 {	svy, subpop(if illness==1): tab `var' did_level, col ci format(%9.3f)	}foreach var of varlist age_child_months health_fac_dist_use num_child_u1 num_child_u5 age {	svy, subpop(if illness==1): mean `var', over(did_level) cformat(%9.1f)}			svy, subpop(if illness==1): mean gb_wealth_index_2018, over(did_level) cformat(%9.2f)		*=======================================================================================*=======================================================================================* SENSITIVITY ANALYSIS: Regression-adjusted model*=======================================================================================*=======================================================================================* Run SVYSET command to apply survey design weights and finite population correctionsvyset clusterid [pweight=weight_sampling_use], strata(strata) fpc(fpc1_use) vce(linearized) singleunit(centered) || id_household, fpc(fpc2_use) * Compositesvy, subpop(if illness == 1): logistic treatment_formal i.lmh_managed##i.survey i.gender_child_health i.gb_wealth_qtile c.health_fac_dist_use i.mother_educ_cat c.age_child_months c.age i.femalelanguage i.illness_gt1 c.num_child_u5 i.mining_communitymargins r.lmh_managed#r.survey, subpop(if illness==1) vce(unconditional)* Feversvy, subpop(if fever_last2wks == 1): logistic treatment_formal i.lmh_managed##i.survey i.gender_child_health i.gb_wealth_qtile c.health_fac_dist_use i.mother_educ_cat c.age_child_months c.age i.femalelanguage i.illness_gt1 c.num_child_u5 i.mining_communitymargins r.lmh_managed#r.survey, subpop(if fever_last2wks==1) vce(unconditional)* Diarrheasvy, subpop(if diarrhea_last2wks == 1): logistic treatment_formal i.lmh_managed##i.survey i.gender_child_health i.gb_wealth_qtile c.health_fac_dist_use i.mother_educ_cat c.age_child_months c.age i.femalelanguage i.illness_gt1 c.num_child_u5 i.mining_communitymargins r.lmh_managed#r.survey, subpop(if diarrhea_last2wks==1) vce(unconditional)* ARIsvy, subpop(if ari_last2wks == 1): logistic treatment_formal i.lmh_managed##i.survey i.gender_child_health i.gb_wealth_qtile c.health_fac_dist_use i.mother_educ_cat c.age_child_months c.age i.femalelanguage i.illness_gt1 c.num_child_u5 i.mining_communitymargins r.lmh_managed#r.survey, subpop(if ari_last2wks==1) vce(unconditional)* ORT for diarrheasvy, subpop(if diarrhea_last2wks == 1): logistic diarrhea_rcvd_ors i.lmh_managed##i.survey i.gender_child_health i.gb_wealth_qtile c.health_fac_dist_use i.mother_educ_cat c.age_child_months c.age i.femalelanguage i.illness_gt1 c.num_child_u5 i.mining_communitymargins r.lmh_managed#r.survey, subpop(if diarrhea_last2wks==1) vce(unconditional)* RDT for feversvy, subpop(if fever_last2wks == 1): logistic fever_rcvd_rdt i.lmh_managed##i.survey i.gender_child_health i.gb_wealth_qtile c.health_fac_dist_use i.mother_educ_cat c.age_child_months c.age i.femalelanguage i.illness_gt1 c.num_child_u5 i.mining_communitymargins r.lmh_managed#r.survey, subpop(if fever_last2wks==1) vce(unconditional)*=======================================================================================*=======================================================================================* SENSITIVITY ANALYSIS: AGRICULTURAL AREAS ONLY*=======================================================================================*=======================================================================================		set seed 39273	gen outcome_random_ag=uniform() if illness==1 & treatment_formal!=. & mining_community==0	// Generates a random variable for balance diagnostics. Sets all obs w/o illness or with missing treatment data to missing. Results in the same number of observations as in the diff-in-diff analyses.				gen outcome_tx_rand_ag=0 if outcome_random<=.5	replace outcome_tx_rand_ag=1 if outcome_random>.5 & outcome_random<.	label var outcome_tx_rand "Random care seeking variable: USE FOR BALANCE DIAGNOSTICS ONLY!"	teffects ipw (outcome_tx_rand_ag) (did_level i.gender_child_health i.gb_wealth_qtile c.ln_distance i.mother_educ_cat c.age_child_months c.age i.femalelanguage i.illness_gt1 c.num_child_u5) if illness==1 & outcome_tx_rand!=. & mining_community==0 [pw=weight_sampling_use] , pomeans 		tebalance sum	predict pscore_tx1_ag pscore_tx2_ag pscore_tx3_ag pscore_tx4_ag if e(sample)==1, ps	gen ipw_tx_ate_ag=1/pscore_tx1_ag if did_level==1	replace ipw_tx_ate_ag=1/pscore_tx2_ag if did_level==2	replace ipw_tx_ate_ag=1/pscore_tx3_ag if did_level==3 	replace ipw_tx_ate_ag=1/pscore_tx4_ag if did_level==4	label var ipw_tx_ate_ag "SENS: AG-ONLY IPTW for childhood treatment analysis"		gen iptw_weight_sens_ag = ipw_tx_ate*weight_sampling_use if ipw_tx_ate != .		label var iptw_weight_sens_ag "SENS: IPT Weight for Agricultural Area Only Analyses"						drop ipw_tx_ate_ag			//1. IPTW models	svyset clusterid [pweight=iptw_weight_sens_ag], strata(strata) fpc(fpc1_use) vce(linearized) singleunit(centered) || id_household, fpc(fpc2_use) 			tab survey if illness == 1 & mining_community==0	svy, subpop(if illness == 1 & mining_community==0): regress treatment_formal i.lmh_managed##i.survey 	* Fever	tab survey if fever_last2wks == 1  & mining_community==0	svy, subpop(if fever_last2wks == 1 & mining_community==0): regress treatment_formal i.lmh_managed##i.survey 		* Diarrhea	tab survey if diarrhea_last2wks == 1 & mining_community==0	svy, subpop(if diarrhea_last2wks == 1 & mining_community==0): regress treatment_formal i.lmh_managed##i.survey 	* ARI	tab survey if ari_last2wks == 1 & mining_community==0	svy, subpop(if ari_last2wks == 1 & mining_community==0): regress treatment_formal i.lmh_managed##i.survey 	* ORT for diarrhea	tab diarrhea_rcvd_ors survey if diarrhea_last2wks == 1 & mining_community==0	svy, subpop(if diarrhea_last2wks == 1 & mining_community==0): regress diarrhea_rcvd_ors i.lmh_managed##i.survey 	* RDT for fever	tab fever_rcvd_rdt survey if fever_last2wks == 1 & mining_community==0	svy, subpop(if fever_last2wks == 1 & mining_community==0): regress fever_rcvd_rdt i.lmh_managed##i.survey 		//2. Unadjusted analyses 		svyset clusterid [pweight=weight_sampling_use], strata(strata) fpc(fpc1_use) vce(linearized) singleunit(centered) || id_household, fpc(fpc2_use) 	* Composite	svy, subpop(if illness == 1 & mining_community==0): regress treatment_formal i.lmh_managed##i.survey 	* Fever	svy, subpop(if fever_last2wks == 1 & mining_community==0): regress treatment_formal i.lmh_managed##i.survey 	* Diarrhea	svy, subpop(if diarrhea_last2wks == 1 & mining_community==0): regress treatment_formal i.lmh_managed##i.survey 	* ARI	svy, subpop(if ari_last2wks == 1 & mining_community==0): regress treatment_formal i.lmh_managed##i.survey 	* ORT for diarrhea	svy, subpop(if diarrhea_last2wks == 1 & mining_community==0): regress diarrhea_rcvd_ors i.lmh_managed##i.survey 	* RDT for fever	svy, subpop(if fever_last2wks == 1 & mining_community==0): regress fever_rcvd_rdt i.lmh_managed##i.survey 	*=======================================================================================*=======================================================================================* SENSITIVITY ANALYSIS: KEEPS DATA EXCLUDED FROM MAIN ANALYSIS FOR QUALITY ASSURANCE REASONS*=======================================================================================*=======================================================================================**!!!!! !!!!! DO NOT USE THIS DATA FOR ANYTHING EXCEPT THE SENSITIVITY ANALYIS**!!!!! !!!!! DO NOT USE THIS DATA FOR ANYTHING EXCEPT THE SENSITIVITY ANALYIS**!!!!! !!!!! DO NOT USE THIS DATA FOR ANYTHING EXCEPT THE SENSITIVITY ANALYIS	preserve		use "lms_gb_2019_child_did_ALL_DATA_FOR SENSITIVITY_DO_NOT_USE.dta", clear		gen ln_distance = ln(health_fac_dist_use)	label var ln_distance "log of distance to health facility"		set seed 12345	gen outcome_random=uniform() if illness==1 & treatment_formal!=.	// Generates a random variable for balance diagnostics. Sets all obs w/o illness or with missing treatment data to missing. Results in the same number of observations as in the diff-in-diff analyses.				gen outcome_tx_rand=0 if outcome_random<=.5	replace outcome_tx_rand=1 if outcome_random>.5 & outcome_random<.	label var outcome_tx_rand "Random care seeking variable: USE FOR BALANCE DIAGNOSTICS ONLY!"		teffects ipw (outcome_tx_rand) (did_level i.gender_child_health i.gb_wealth_qtile c.ln_distance i.mother_educ_cat c.age_child_months c.age i.femalelanguage i.illness_gt1 c.num_child_u5) if illness==1 & outcome_tx_rand!=. [pw=weight_sampling_use] , pomeans 		tebalance sum		predict pscore_tx1 pscore_tx2 pscore_tx3 pscore_tx4 if e(sample)==1, ps	gen ipw_tx_ate=1/pscore_tx1 if did_level==1	replace ipw_tx_ate=1/pscore_tx2 if did_level==2	replace ipw_tx_ate=1/pscore_tx3 if did_level==3 	replace ipw_tx_ate=1/pscore_tx4 if did_level==4	label var ipw_tx_ate "IPTW for childhood treatment analysis"		gen iptw_weight_sens = ipw_tx_ate*weight_sampling_use if ipw_tx_ate != .		**!!!!! !!!!! DO NOT USE THIS DATA FOR ANYTHING EXCEPT THE SENSITIVITY ANALYIS	**!!!!! !!!!! DO NOT USE THIS DATA FOR ANYTHING EXCEPT THE SENSITIVITY ANALYIS	**!!!!! !!!!! DO NOT USE THIS DATA FOR ANYTHING EXCEPT THE SENSITIVITY ANALYIS		svyset clusterid [pweight=iptw_weight_sens], strata(strata) fpc(fpc1_use) vce(linearized) singleunit(centered) || id_household, fpc(fpc2_use) 			//1. IPT weighted analyses	*Composite	tab survey if illness == 1	svy, subpop(if illness == 1): regress treatment_formal i.lmh_managed##i.survey 	* Fever	tab survey if fever_last2wks == 1	svy, subpop(if fever_last2wks == 1): regress treatment_formal i.lmh_managed##i.survey 		* Diarrhea	tab survey if diarrhea_last2wks == 1	svy, subpop(if diarrhea_last2wks == 1): regress treatment_formal i.lmh_managed##i.survey 	* ARI	tab survey if ari_last2wks == 1	svy, subpop(if ari_last2wks == 1): regress treatment_formal i.lmh_managed##i.survey 	* ORT for diarrhea	tab diarrhea_rcvd_ors survey if diarrhea_last2wks == 1	svy, subpop(if diarrhea_last2wks == 1): regress diarrhea_rcvd_ors i.lmh_managed##i.survey 	* RDT for fever	tab fever_rcvd_rdt survey if fever_last2wks == 1	svy, subpop(if fever_last2wks == 1): regress fever_rcvd_rdt i.lmh_managed##i.survey 	**!!!!! !!!!! DO NOT USE THIS DATA FOR ANYTHING EXCEPT THE SENSITIVITY ANALYIS	**!!!!! !!!!! DO NOT USE THIS DATA FOR ANYTHING EXCEPT THE SENSITIVITY ANALYIS	**!!!!! !!!!! DO NOT USE THIS DATA FOR ANYTHING EXCEPT THE SENSITIVITY ANALYIS		//2. Unadjusted analyses 		svyset clusterid [pweight=weight_sampling_use], strata(strata) fpc(fpc1_use) vce(linearized) singleunit(centered) || id_household, fpc(fpc2_use) 	* Composite	svy, subpop(if illness == 1): regress treatment_formal i.lmh_managed##i.survey 	margins r.lmh_managed#r.survey, subpop(if illness==1) vce(unconditional)	* Fever	svy, subpop(if fever_last2wks == 1): regress treatment_formal i.lmh_managed##i.survey 	margins r.lmh_managed#r.survey, subpop(if fever_last2wks==1) vce(unconditional)	* Diarrhea	svy, subpop(if diarrhea_last2wks == 1): regress treatment_formal i.lmh_managed##i.survey 	margins r.lmh_managed#r.survey, subpop(if diarrhea_last2wks==1) vce(unconditional)	* ARI	svy, subpop(if ari_last2wks == 1): regress treatment_formal i.lmh_managed##i.survey 	margins r.lmh_managed#r.survey, subpop(if ari_last2wks==1) vce(unconditional)	* ORT for diarrhea	svy, subpop(if diarrhea_last2wks == 1): regress diarrhea_rcvd_ors i.lmh_managed##i.survey 	margins r.lmh_managed#r.survey, subpop(if diarrhea_last2wks==1) vce(unconditional)	* RDT for fever	svy, subpop(if fever_last2wks == 1): regress fever_rcvd_rdt i.lmh_managed##i.survey 	margins r.lmh_managed#r.survey, subpop(if fever_last2wks==1) vce(unconditional)		**!!!!! !!!!! DO NOT USE THIS DATA FOR ANYTHING EXCEPT THE SENSITIVITY ANALYIS	**!!!!! !!!!! DO NOT USE THIS DATA FOR ANYTHING EXCEPT THE SENSITIVITY ANALYIS	**!!!!! !!!!! DO NOT USE THIS DATA FOR ANYTHING EXCEPT THE SENSITIVITY ANALYIS	restore	
