# Supplementary material for: Impact of the Liberian National Community Health Assistant Program on childhood illness care in Grand Bassa County, Liberia
Source: PLOS Glob Public Health. 2022 Jun 30;2(6):e0000668. doi: 10.1371/journal.pgph.0000668 (PMC10021826; doi:10.1371/journal.pgph.0000668)
Supplement: S1 Text — (DOCX) [file pgph.0000668.s005.docx]

Inclusivity in global research

PLOS’ policy on inclusivity in global research aims to improve transparency in the reporting of research performed outside of researchers’ own country or community and ensures that PLOS publications reporting global research adhere to high standards for research ethics and authorship. Authors of relevant research articles may be asked to complete the questionnaire below, which outlines ethical, cultural, and scientific considerations specific to inclusivity in global research. This questionnaire may be requested when researchers have travelled to a different country to conduct research, if research uses samples collected in another country, research with Indigenous populations or their lands, or if research is on cultural artefacts. Researchers travelling to another country solely to use laboratory equipment will not normally be required to complete the questionnaire. However, the questionnaire can be requested at the journal’s discretion for any submission – if you have been requested to complete this questionnaire by the PLOS journal you submitted to, please do so.

Please complete the questionnaire below and include this as a Supporting Information file with your manuscript. Note that if your paper is accepted for publication, this checklist will be published with your article in the supporting information files. Please ensure that you reference the checklist in the main body of your manuscript. We suggest adding a subsection ‘Inclusivity in global research’ to your Methods section and adding the following sentence: “Additional information regarding the ethical, cultural, and scientific considerations specific to inclusivity in global research is included in the Supporting Information (SX Checklist)”

The questions have been designed to be applicable to a wide range of study types, and there are subsections for both human subjects research and non-human subjects research. If any of the questions are not relevant to your research please mark them as “N/A” as appropriate.

**Ethical considerations, permits and authorship**

*This section is applicable to all research types.*

Provide details as to who granted permissions and/or consent for the study to take place in the Methods section of your manuscript. This should include the names of **all** ethics boards, governmental organizations, community leaders or other bodies that provided approval for the study. If individuals provided approval refer to these people by their role or title but do not list their name(s).

Reported on page number: page 11

If there were any deviations from the study protocol after approval was obtained please provide details of these changes in the Methods section of your manuscript.
Did this study involve local collaborators that are residents of the country where the research was conducted or members of the community studied? If you do not have any authors from said communities, please provide an explanation for this below.

Reported on page number: Not applicable

Yes. SM, FRK, RK, BG, GPJ, and MS are residents of Liberia.

Everyone listed as an author should meet PLOS’ criteria for authorship and all individuals who meet these criteria should be included in the author byline, rather than the acknowledgements. Authorship criteria is based on the International Committee of Medical Journal Editors (ICMJE) Uniform Requirements for Manuscripts Submitted to Biomedical Journals - for further information please see here: <https://journals.plos.org/plosone/s/authorship>.

**Human subjects research (e.g. health research, medical research, cross-cultural psychology)**

Did you obtain written informed consent from a representative of the local community or region before the research took place? How did you establish who speaks for the community? Details of written informed consent obtained from study participants should be reported separately in the Methods section of your manuscript.

We discussed the study with both the Ministry of Health and County Health teams, which are the relevant governmental entities with jurisdiction over the population in which we work. Both approved the activities (and authors from both are included on this paper). Prior to surveying in a community, the surveyors meet with the leader of that community to explain the survey and receive permission to conduct the survey. We do not receive this permission in writing, in part because the primary language where we conduct surveys does not have a commonly used written form.

How did members of the local community provide input on the aims of the research investigation, its methodology, and its anticipated outcome(s)?

We fully engaged relevant officials of the Ministry of Health, Grand Bassa County Health Team from the beginning of the study (including study design, analysis, and presentation of results). The Ministry of Health conducts ongoing qualitative work to understand how the National Community Health Assistant Program is perceived by key community-level stakeholders, and Last Mile Health supports the conduct of this work. The intervention being evaluated is designed, owned, and led by the Ministry of Health.

When engaging with the local community, how did you ensure that the informed consent documents and other materials could be understood by local stakeholders?

We took several steps to ensure that consent forms could be understood so that consent was fully informed. First, Liberian staff developed the English forms to make sure that local terminology was used correctly, and these forms were approved by the in-country IRB (which is the lead IRB for this study), as well as the US-based IRBs. All surveyors were bilingual in Bassa so that respondents who only spoke Bassa could be consented in their preferred language. Bassa does not have a commonly used written form, so these materials could not be written, but we worked extensively with surveyors to ensure that they were providing information fully and receiving informed consent. The IRBs signed off these procedures.

Will the findings of the research be made available in an understandable format to stakeholders in the community where the study was conducted (e.g. via a presentation, summary report, copies of publications, etc.)? Please provide details of how this will be achieved.

The findings have been shared with relevant officials at the Ministry of Health and the County Health Team, representatives of which are authors on this study. As further data are collected, we will continue sharing results in presentations and written reports.
