# Supplementary material for: Impact of the Liberian National Community Health Assistant Program on childhood illness care in Grand Bassa County, Liberia
Source: PLOS Glob Public Health. 2022 Jun 30;2(6):e0000668. doi: 10.1371/journal.pgph.0000668 (PMC10021826; doi:10.1371/journal.pgph.0000668)
Supplement: Attachment — Submitted filename: Response to Reviewers 22feb2022.docx [file pgph.0000668.s006.docx]

*Many thanks for the careful review of our manuscript and the opportunity to submit a revised version of it. Below, we respond to the editors’ and reviewers’ comments and describe edits we have made to the manuscript. For ease of reading, the reviewers’ feedback are in black plainface type, and our responses are in blue italics.*

Journal Requirements:

1. Please include a complete copy of PLOS’ questionnaire on inclusivity in global research in your revised manuscript. Our policy for research in this area aims to improve transparency in the reporting of research performed outside of researchers’ own country or community. The policy applies to researchers who have travelled to a different country to conduct research, research with Indigenous populations or their lands, and research on cultural artefacts. The questionnaire can also be requested at the journal’s discretion for any other submissions, even if these conditions are not met. Please find more information on the policy and a link to download a blank copy of the questionnaire here: <https://journals.plos.org/plosone/s/best-practices-in-research-reporting>. Please upload a completed version of your questionnaire as Supporting Information when you resubmit your manuscript.

*We have completed this questionnaire and uploaded it as supporting information.*

2. We have noticed that you have uploaded supporting information but you have not included a list of legends. Please add a full list of legends for all supporting information files (including figures, table and data files) after the references list.

*Many thanks. We have provided a list of legends for all supporting information files.*

3. In the online submission form, you indicated that "De-identified data are available from the authors upon reasonable request.". All PLOS journals now require all data underlying the findings described in their manuscript to be freely available to other researchers, either 1. In a public repository, 2. Within the manuscript itself, or 3. Uploaded as supplementary information.
This policy applies to all data except where public deposition would breach compliance with the protocol approved by your research ethics board. If your data cannot be made publicly available for ethical or legal reasons (e.g., public availability would compromise patient privacy), please explain your reasons by return email and your exemption request will be escalated to the editor for approval. Your exemption request will be handled independently and will not hold up the peer review process, but will need to be resolved should your manuscript be accepted for publication. One of the Editorial team will then be in touch if there are any issues.

*Apologies for misunderstanding the journal policy. We have uploaded replication data for the study to Mendeley Data, where it is publicly available at* [*https://data.mendeley.com/datasets/7h9f9mkh9b/1*](https://data.mendeley.com/datasets/7h9f9mkh9b/1)*. We have uploaded replication code as a supplement. We have updated the data availability statement on Editorial Manager accordingly.*

4. Please ensure that the details in the Funding Information is the same with Financial Disclosure Statement.

*Many thanks. We have updated the financial disclosure statements in light of an organization-wide review of disclosure best practices. As a result, we are disclosing funders who support research but not funders who only provided general funding to Last Mile Health overall. The belief of the organization is that the lengthy list of funders who provided organizational funding dilutes understanding of who funded the research activities. We have double-checked that the details are the same in the Funding Information and Financial Disclosure Statement. No funders had input into the design, data interpretation of the study or manuscript.*

Reviewer #1:

1. Infant survival is an important investment that can be made by any health system care delivery. A 15% reduction in Under-five mortality recorded in the country under review is justification warranting health system strengthening to sustain gains recorded. The observed weaknesses in the domain of reinforcing and enabling factors is accountable for poor health outcomes for children of under-five years in the country and training community health workers through the implementation of a pilot community-based training program is considered a strategy of overcoming system weaknesses.

*Many thanks. We agree that that well trained, community-based health workers is an important strategy for overcoming challenges in health systems in remote areas.*

2. The title needs modification; “Impact of the Liberian National Community Health Assistant Program on Childhood Illness-Treatment outcomes in Grand Bassa County, Liberia”

*Apologies, but we are not able to shorten the title in this way because our understanding of the journal style guide is that it requires a brief discussion of the basic research design.*

3. The research questions may be better written as; Line 119 should read; To what extent would the intervention programme produce changes in child health treatment outcomes between implementation area over control?

*We agree that we wrote this confusingly and made edits to the first research question for clarity.*

4. Overall Observations: There is observed lack of recourse to any theoretical clarifications underpinning health promotion principles and public health principles of prevention and control in the developing the programme and implementation. This translates to a superficial study devoid of theoretical foundation.

*This article is an evaluation of a national program developed and led by the Liberia Ministry of Health in response to lessons learned from data and other CHW program evidence in Liberia and other countries and during the Ebola virus outbreak where trust and access was related to presence of CHWs. The program also reflects learning from core principles around quality (access, people-centeredness, equity). As such, we would respectfully disagree with the contention that there is no theoretical foundation of our study, which is based on solid principles of program design, epidemiology, and statistical analysis. See, for example, references 8-10, which go into detail about the design of such programs in general.*

*The development of the program in Liberia has been extensively described in previous papers. In particular, we would note references 13, 14, and 16 describe the Liberian National CHA Program and the justifications for its design in detail. References 11 and 12 describe results from various precursor programs to the National CHA Program, which provide support for the hypothesis that this program would be effective.*

Since the problem phenomenon has predominantly two sources of challenges, strategic planning should evolve from diagnostic considerations of the reinforcing and enabling factors underpinning the problem phenomenon. In the process of designing the study, there are no evidence of applying theoretical resources that would guide elucidation of proof of concept. This grossly overlooked application of public health science in the planning and implementation of the study constitute weakness this type of study cannot afford. Secondly, personal-level dispositions of the target population for the intervention, the CHW, have not been considered. These observed lack of theoretical grounding makes the study unscientific and flawed.
In such intervention studies, behaviour models and theories play significant role in providing theoretical-grounding to accommodate predisposing, Reinforcing and Enabling factor considered to be responsible for the problem phenomenon so that the programme developed is based on adequate appropriate diagnostic principles and not superficially assuming that a training package would address the issues responsible for the problems appropriately.

*While we agree that often public health programs are not designed from a clear process of “diagnostic considerations of the reinforcing and enabling factors underpinning the problem phenomenon”, this was not the scope or goal of our study, which was instead a programmatic evaluation of a national program to inform current and future work to increase access and quality needed to meet the health-related SDG-goals and UHC. As noted above, we have included references which discuss the diagnosed challenges which the CHA program is designed to address. Further, we note that our study provides robust evidence that the Liberian National CHA Program is, in fact, producing substantial benefits—which would tend to substantiate the programmatic theory and justifications.*

-Statistical analysis is completely flawed. In such pre- and post-test intervention programmes, scores are generated for the target samples (Experimental group and Control group), this was not the case here, rather frequency distribution was used, whereas a paired-sample t-test is most appropriate with a computation of Mean-based Effect Size to determine the magnitude or impact of the intervention.

*We appreciate this feedback, but we respectfully disagree with the suggestion to use paired-sample t-tests. The paired-sample t-test is appropriate when (1) the same individuals are being measured at the pre- and post-intervention periods and (2) endpoint of interest is measured with a continuous variable. In our study, neither criteria applies. Our data are from repeated cross-sectional samples – that is, the data are not of the same individuals at different time points, but rather representative samples of the same population (but with different individuals) at the time points. Further, our outcomes are proportions, not continuous variables. Our statistical approach is customary for this type of data and research question. See, for example, the references by Stuart et al (*[*https://pubmed.ncbi.nlm.nih.gov/25530705/*](https://pubmed.ncbi.nlm.nih.gov/25530705/)*) for a methodological overview and see White et al (*[*https://pubmed.ncbi.nlm.nih.gov/30024811/*](https://pubmed.ncbi.nlm.nih.gov/30024811/)*) and Nyblade et al (*[*https://www.ncbi.nlm.nih.gov/pmc/articles/PMC7180216/*](https://www.ncbi.nlm.nih.gov/pmc/articles/PMC7180216/)*) for examples of previous use to address analogous research questions.*

5. If the authors are able to address the lack of theoretical and conceptual resources as basis for developing the intervention programme and data analysis, then the weaknesses in the study would have been ameliorated.

Reviewer #2:

1. Abstract, paragraph 2. The statement “we measured before-to-after changes in childhood treatment from qualified providers” is too broad. This sentence does not give any indication about what issues related to childhood are being addressed. Suggest you modify to “… changes in treatment of common childhood illnesses from qualified providers…” *Many thanks – we have edited accordingly.*

2. Abstract, paragraph 2. It is not informative to have percentage increase in your primary outcome in the abstract without any indication of the scale or denominator of your population. It could be an increase among 10 children or among 100,000 people. Please mention the total number of participants in your surveys. *Many thanks. We have added that there were a total of 1291 children with childhood illnesses included in the analysis.*

3. Introduction, line 78. Please clarify that this is “all-cause under five mortality” *We made this edit.*

4. Introduction, line 82. Please either use the standardized term “all-cause under-five mortality”, or specify what causes of death you describing (e.g. only infectious disease deaths). *Many thanks. We clarified that we are referring to all-cause under-five mortality.*

5. Introduction, line 92. Describing this study as “proof of principle” suggests that you’re conducting a very controlled efficacy study, rather than what I assume is a effectiveness study exploring the impact of the program in a more routine implementation setting. Please clarify.

*Many thanks. This is a fair point. The first iteration of the program, launched in 2012, was a fairly exploratory endeavor. We have revised the language to clarify this and to remove the “proof of principle” text which you rightly point out suggests a more tightly controlled efficacy study than what was done at that stage.*

6. Introduction lines 94-99. Further to my previous comment, if there are already two published evaluations of this project, why is this analysis considered the proof of principle? How does this paper add to what’s already published, or is each county just getting a separate publication? Is this a larger scale evaluation with more data / areas included? Used a different implementation / support module? MoH led rather than partner agency?

*We have added clarity here – our original text did not do a very good job of explaining the iterations. The first two studies that we discussed were iterations of a primarily NGO-led intervention (albeit in close communication with MOH). The current study evaluates the subsequent program, which is MOH-led rather than NGO-led. We made minor edits to these paragraphs to clarify the evolution and highlight that the current iteration is under a ministry-led implementation model. To the best of our knowledge, this is the first study that seeks to evaluate the ministry-led model adopted via the national community health assistant program.*

7. Introduction line 106-7. This suggests your current analysis will evaluate the full national program. Why was this not done, for example using a stepped wedge to account for the phased roll-out of the program?

*We very much wish that a country-wide evaluation were feasible, and we agree that an observational stepped wedge that leverages phased implementation in different parts of the country would be ideal. Unfortunately, there are not population-based data sources that cover the full geographic range of the country staggered at the right intervals to align with the phases of implementation. (We had intended something akin to this using Demographic and Health Survey data, but the most recent Liberian DHS deleted questions about care from community health workers and did not line up well with phases of implementation, so using those data proved not to be feasible.) The analysis was feasible in Grand Bassa County because Last Mile Health administers annual population-based surveys, which could be aligned with implementation in that county – but not in counties with other implementing partners.*

8. Introduction, line 114-5. Need to also mention the key DiD assumption that intervention & comparison areas are assumed to be otherwise similar. *Many thanks. We have added text that the assumption of our design is that comparison area trends represent what would have occurred in intervention areas if they had not received the intervention. We later discuss in the methods section how we used inverse probability of treatment weighting to make the intervention and comparison areas as comparable as possible.*

9. Methods. Some additional information is required to clarify the county mapping and sampling procedures.
a. Did you conduct a complete census of every household in the study counties by sending teams out to visit all houses? Or get estimated number of households from local leaders or administration, or use existing government national census data?

*Prior to the baseline survey, we constructed a sampling frame by mapping every community in the county and taking a complete enumeration of households in each. (The most recent census data in Liberia is from 2008, so it is no longer usable as a sampling frame.) We have updated the text to clarify this and made a few minor edits in the supplement to further clarify.*

b. Please present your sample size assumptions to confirm that you had “adequate precision over the planned series of surveys”

*We powered the surveys to estimate childhood disease treatment within five percentage points, and we have added this to the methods section.*

c. What was the modified random walk? Please include a couple of sentences explaining how this was done.

*Please see below – we discuss this in more detail in response to a subsequent query. We have added more description to the methods supplement.*

d. What indicators did you use to ensure intervention and comparator districts were comparable on baseline health indicators? Did you also include these indicators in your IPW or multivariable models?

*Comparison districts were chosen as the most similar districts which had not yet implemented, based primarily on health outcomes (e.g. childhood illness treatment, childhood vaccination coverage, antenatal care, facility-based delivery, etc). We did not include these factors in IPTW and regression models because they are outcomes of the intervention so inclusion would be inappropriate. However, we do present baseline differences in the outcomes we are studying in this study in figure 1, and the baseline values are quite similar.*

e. How did you sample based on PPS unless you have a complete census?

*Many thanks. We did have a complete census for our sampling frame taken at baseline. We did not update the complete enumeration of households in the county at follow-up because the follow up survey was only conducted about 12 months after the original census. We use the guidance from UN Stats that censuses taken within two years are suitable for sampling frames (e.g. page 91 of* [*https://unstats.un.org/unsd/demographic/sources/surveys/handbook23june05.pdf*](https://unstats.un.org/unsd/demographic/sources/surveys/handbook23june05.pdf)*) and so we only take a full enumeration if there aren’t reliable population total from within that period. We have made minor edits to the methods supplement to discuss this in more detail. We have also changed the description of the follow-up sample to probability proportional to estimated size to account for the one-year lag since the census.*

10. Methods, line 178. I’m curious why your primary outcome of interest is receiving treatment from a qualified provider, rather than just seeing care from a qualified provider. Particularly for children with suspected malaria or suspected pneumonia, treatment should only be provided if the child meets the criteria stated in national guidelines (e.g. positive malaria rapid test). Does this mean that children seeking care but who didn’t receive any drugs are excluded from the analysis?

*Many thanks. This outcome is based on the following question (which is in Liberian vernacular English and is repeated for each illness: “You got treatment from anywhere or anyone the time [of fever/ARI/diarrhea]?” In vernacular English, “treatment” connotes interaction with the provider but not necessarily a specific treatment – for example, receipt of RDT would be reported even if the RDT was negative and there was no further treatment. However, it is more than just seeking care in that it connotes an actual care interaction with the provider. We have updated “treatment” with “care” throughout to clarify this.*

11. Results, line 241. Does the 2538 households in 2018 include participants from counties that weren’t included in the final DiD analysis? If it was just the counties included in the analysis, can you explain why the sample size is so much smaller in 2019?

*The analysis samples are of the same populations at both baseline and follow-up. We drew a substantially larger sample at baseline for programmatic reasons. The principal reason for the baseline sample was to help plan the intervention, so it was powered to outcomes that affected a smaller part of the population (e.g. vaccination for children 12-23 months old) and to be able to disaggregate more extensively than the follow-up survey.*

12. Discussion, line 313. If you are proposing that your results can be interpreted causally, I suggest providing further justification from the evaluation literature that your analysis approach and the implementation environment are suitable to make these causal conclusions.

*Many thanks. We do believe it appropriate to make a causal inference from our findings. We have added a paragraph to the discussion section describing why we believe this inference is warranted. We cite to and discuss both the difference-in-differences literature and the inverse probability of treatment weighting literature, including methods papers that show that a well-formulated IPTW model can mitigate against the risk different pre-intervention trends (which we cannot directly assess because we only have one pre-intervention time period).*

13. Discussion lines 314-315. Is this availability of ORS and RDTs at trained health providers (e.g. lack of stock outs) or receipt of these commodities by eligible individuals?

*We intended this to mean the receipt of these commodities and have edited that sentence to clarify.*

14. Discussion, line 356. I find the equity by distance principle a little hard to understand, since the CHA program is presumably targeted to the communities furthest from health facilities. Did you look at distance from a qualified care provider after the CHA program was rolled out, or before? Is this question actually asking about whether CHAs were targeted to the most in-need communities?

*Many thanks. In this part of the discussion, we were aiming to highlight that there is often heterogeneity in access to care by relative remoteness even within populations that are entirely remote. We agree that the CHA program is designed to overcome this challenge. However, community health worker programs are more difficult to operate well in the more remote communities in which they operate – for example, supply chains are harder to maintain in locations with the poorest road infrastructure and supervision may suffer when supervisors have to make lengthier trips to CHAs. As a result, we believe it is important to see if the CHA program is sufficing to close outcome gaps in the more highly remote communities being served. We have made minor edits to clarify this and to clarify that we are measuring relative distance from health facilities (which did not change over the program period).*

15. Discussion, line 377. Suggest change “resulted in” to “associated with” unless you strengthen your justification that this is a causal effect analysis.

*Many thanks. We strengthened our justification for causal inference as noted above.*

16. Discussion, line 380-1. I didn’t see any data on quality of care, only that individuals were attending qualified rather than unqualified health providers. Did you assess the quality of care of CHAs compared to a sample of the unqualified providers?

*Thanks – we intended this to mean that patients were more likely to receive essential elements of care (ORT and malaria rapid diagnostic tests) and edited this sentence to clarify that.*

17. In the discussion, it would be interested to hear if you anticipate these levels of usage of CHAs to remain stable over time, since the second surveys was only 5 months after the intervention roll out, do you anticipate these levels of usage of CHAs to remain stable over time? Is there any experience from the earlier pilots in Liberia?

*We do expect that these levels will be sustained over time, though we do not yet have direct evidence on this. The main impediment is that we stopped our surveys due to covid and only recently resumed surveying. So, while we have preliminary data showing that current levels of CHA usage are similar to what we report here (from other counties in Liberia), we have not had the opportunity to fully analyze those data. One study that we cite in the discussion (Rozelle et al, reference 26) provides evidence that CHAs continued to provide an increasing proportion of malaria care early in the covid epidemic based on CBIS data.*

*We are a little hesitant to speculate about how levels have persisted after the study period in the manuscript because we do not have direct data yet and because covid has complicated interpretations, but we do note in the discussion that assessing this is one goal of a survey we are running later this spring.*

18. Figure 1. Please add labels to the y-axes.

*We have added axis labels.*

19. Figure 2. Percent of children receiving what treatment? Or do you mean seeking care from a qualified health provider in the last 2 weeks?

*We have retitled this figure to clarify that it is receipt of care from each of the various provider types.*

20. Figure 3. As previous comment. Treatment of what?

*Many thanks. We have retitled this figure to clarify that it refers to care from a qualified provider.*

21. Supplement 1. Sampling. You mention in the main article using PPS, but here say it was a simple random sample. I am also still unclear about how you did PPS or the simulations mentioned here without further information on whether county-wide census was performed. Furthermore, if you had a full census for the sampling, why did you then use random walk to select households – which may result in some geographic biases – rather than a random selection from your census data?.

*Many thanks for raising this. We used different sampling schemes at baseline and follow-up, but both permit population-representative estimates. At baseline, we conducted a simple random sample of communities and then surveyed everyone in selected communities. At follow-up we conducted the more common PPES sample of communities and then took a fixed sample (24 households) per community. As described above, we did conduct a full census prior to the baseline survey, which was still appropriately current at follow-up.*

*We have debated the merits of a random walk approach versus pre-selection of specific households on our team. The trade-offs as we perceive them are between (1) the possibility of geographic bias that you mention from random walk and (2) easier supervision of adherence to selection with the random walk. Our survey implementation managers believe that the latter outweighs the former, at least in our setting. We would note that we believe the risk of geographic bias to be small for two reasons. First, the modification we use which starts the random walk at multiple points and at a random starting point between the center and perimeter of the community for each should result in broader dissemination of sampled households per community (a technique recommended by Bennett et al). Second, the communities we are sampling are very small – usually only a couple dozen households. As a result, communities are fairly homogenous and we end up sampling a very high fraction of selected communities. This is consistent with prior research which suggests bias from random walk procedures are more of an issue in urban areas than rural areas. We have added more detail to the supplement to detail this more completely.*

22. Supplement 1. IPTW. What is meant by the number of illnesses? Should that have been included in the IPW considering it is independently associated with outcomes relating to treatment?

*By this we meant whether the parent reported symptoms suggesting more than one childhood condition (e.g. fever and diarrhea). We included it in the IPTW models because we believe it could be a proxy for unmeasured social disadvantage and thus possibly a proxy for a confounder. It is possible that it could be downstream from health promotion activities CHAs conduct, but we believe that to be a lesser likelihood.*

*In response to your query, we ran IPTW models that excluded this variable to see if it meaningfully affected the results. The difference-in-difference estimates do not meaningfully change without it in the model: 55.2 percentage points (p<0.001) for care from a qualified provider for all illnesses; 60.7 percentage points (p<0.001) for fever care from a qualified provider; 69.8 percentage points (p<0.001) for diarrhea care from a qualified provider; 39.1 percentage points (p=0.007) for ARI care from a qualified provider; 32.9 percentage points (p<0.001) for ORT receipt; and 41.9 percentage points (p<0.001) for RDT receipt.*
