# Supplementary material for: Impact of the Liberian National Community Health Assistant Program on childhood illness care in Grand Bassa County, Liberia
Source: PLOS Glob Public Health. 2022 Jun 30;2(6):e0000668. doi: 10.1371/journal.pgph.0000668 (PMC10021826; doi:10.1371/journal.pgph.0000668)
Supplement: Attachment — Submitted filename: Response to Reviewers.docx [file pgph.0000668.s007.docx]

*Many thanks for the opportunity to revise this manuscript. We have made a number of revisions in response to the reviewers’ feedback, as well as several clarifications.. In one instance, we have declined to make the change because the statistical analysis the reviewer is requesting is not mathematically valid. We have detailed this in our response below. For ease of reading, we have left reviewer feedback in black plainface type and our responses in blue italics.*

4. Have the authors made all data underlying the findings in their manuscript fully available (please refer to the Data Availability Statement at the start of the manuscript PDF file)?

The [PLOS Data policy](https://journals.plos.org/globalpublichealth/s/data-availability) requires authors to make all data underlying the findings described in their manuscript fully available without restriction, with rare exception. The data should be provided as part of the manuscript or its supporting information, or deposited to a public repository. For example, in addition to summary statistics, the data points behind means, medians and variance measures should be available. If there are restrictions on publicly sharing data—e.g. participant privacy or use of data from a third party—those must be specified.

Reviewer #1: No

Reviewer #2: Yes

Reviewer #3: No

*Many thanks. We have uploaded the data that underlie this study to Mendeley data, where it is freely available. The updated data access statement now reads “Replication data are available at https://data.mendeley.com/datasets/7h9f9mkh9b/1. Statistical code to replicate analyses are included as a supplement to this manuscript.”*

6. Review Comments to the Author

Reviewer #1: All recommendations suggested in the first round of review to strengthen the manuscript were totally ignored.

*We are sorry that the reviewer feels we ignored all recommendations, which was very much not our intent. We attempted to respond to all of them, with the exception of one recommendation where we believe the requested revision is mathematically invalid. We will explain our further edits and rationale below.*

The following recommendation where made:
1. The observed weaknesses in the domain of reinforcing and enabling factors is accountable for poor health outcomes for children of under-five years in the country and training community health workers through the implementation of a pilot community-based training program is considered a strategy of overcoming system weaknesses. THESE WERE NOT CONSIDERED IMPORTANT SOURCE OF THE PROBLEM TO BE ESTABLISHED AS FOUNDATIONAL PRINCIPLES TO JUSTIFY THE INTERVENTION.

*Many thanks for this comment. We had previously noted that this is an evaluation of the Liberian national community health assistant program, which is designed specifically to account for geographic and income barriers to care among rural populations in Liberia. The program design and theory is fully described in references that we have cited. However, it is a fair point that one might train a cadre of community health workers but not adequately reinforce that training or meet the preconditions that enable them to meet community needs. We have added text in the background section that better explains that the overall program design was theory-based and corresponds to the current evidence of best practices in community health worker program design. We also added text to explain that the program is designed to overcome the principle barriers to care observed by the Liberian government in this population and that the program uses a supervision strategy that continues to reinforce skills learned in the training. We cite to several published articles that fully explain the theory and logic of the national community health assistant program in more detail than we can in this paper presenting its evaluation.*

2. The title needs modification; “Impact of the Liberian National Community Health Assistant Program on Childhood Illness-Treatment outcomes in Grand Bassa County, Liberia” NO CHANGE IN THE TITLE RECOMMENDED, WAS MADE.

*We appreciate this feedback and have removed “a difference in difference analysis of population-based data” from the title. We had interpreted the journal’s instruction to authors to require including that information, but if it is not required we agree that simplicity is better.*

3. To what extent would the intervention programme produce changes in child health treatment outcomes between implementation area over control? NOT EFFECTED.

*Many thanks for highlighting this. We disagree that this has not been addressed. Rather, we would note that our estimated treatment effect, a difference-in-differences in care from a qualified provider is a measure of greater improvement in outcomes in the intervention area compared to comparison areas. That is, it measures the difference in improvements: the improvement in the intervention area minus the improvement in the comparison area, with the latter representing the best estimate of the counterfactual improvement that would have happened without the intervention. We present these changes compared to the comparison areas in the text, in table 2, and in figure 1.*

4. Statistical analysis is completely flawed. In such pre- and post-test intervention programmes, scores are generated for the target samples (Experimental group and Control group), this was not the case here, rather frequency distribution was used, whereas a paired-sample t-test is most appropriate with a computation of Mean-based Effect Size to determine the magnitude or impact of the intervention. Currently, global best practices for before-and-after intervention would quantify changes as Effect Size, which was recommended but not considered.

*We appreciate the reviewer’s feedback on the statistical analysis, but we strongly disagree that it is completely flawed. While there are studies for which a paired-sample t-test is the appropriate statistical test to and the effect size is thus estimated as a difference in means, that analysis does not align with the research design we employed. We apologize that our explanation of this was insufficient clear in our last response and we will aim to clarify it better in this response.*

*We suspect that the reviewer is basing this critique on methods resources that recommend a paired-sample t-test for before-after evaluations that (1) do not have a comparison group and (2) have outcomes measured on a continuous scale, (3) where the exact same participants are in the before-intervention and after-intervention sample, and (4) where no further adjustment for confounders would be useful.. Those are the conditions under which such an analysis is appropriate. The recommended analysis is not, however, appropriate for our study for several reasons.*

*First, no t-test, whether using paired or independent samples, is appropriate with dichotomous outcomes such as ours. T-tests are only appropriate with continuous outcomes, such as if an intervention is designed to reduce blood pressure and the outcome of interest is mean blood pressure before and after the intervention. Second, paired tests (whether t-tests for continuous data or tests such as McNemar’s for dichotomous data) are only appropriate if the same participants are included in the before and after samples. In our study, as is best practice for evaluating this type of intervention, the same participants are not enrolled at both time points. Rather, we take representative samples of the population at both time points. This allows us to avoid the serious bias that would likely result from attrition over time in this population.*

*Finally, t-tests, while appropriate for a simple before-after comparison are not appropriate for the more complicated analyses our evaluation requires. Our evaluation is not merely a comparison of two groups’ outcomes: before and after the intervention. Rather, four values must be incorporated: before and after in the intervention areas as well as before and after in the comparison areas. This requires analysis of the difference in before-to-after changes in intervention compared to control, which is not feasible with t-tests. Further, our analysis incorporates adjustment for time-varying confounding, which is also not feasible with t-tests.*

*For all these reasons, it is not appropriate to use the paired-sample t-tests that the reviewer has recommended. The approach that we used is a standard approach for evaluating interventions where the outcome (care from a qualified provider) is measured dichotomously in an intervention group compared to a comparison group. The standard approach for this kind of analysis is to fit a linear regression in which one regresses the outcome on an indicator variable for intervention versus comparison, and indicator variable for before versus after, and the interaction of those variables, and with robust standard errors, confidence intervals and p-values are unbiased even though the outcome is dichotomous rather than the continuous outcomes normally used with linear regression. We then further adjusted for confounders that may change differently over time in the intervention areas than the comparison areas using inverse probability of treatment weighting. This approach to adjusted difference-in-differences analysis is well supported in the literature; see for example an excellent methods overview by Elizabeth Stuart at Johns Hopkins University that is available at* [*https://pubmed.ncbi.nlm.nih.gov/25530705/*](https://pubmed.ncbi.nlm.nih.gov/25530705/) *and which we have cited.*

*We are confused by the reviewer’s statement that “global best practices for before-and-after intervention would quantify changes as Effect Size, which was recommended but not considered.” First, our design is not a before-after analysis (which would only incorporate the intervention group and not the comparison group). Second, “effect size” is simply a general term for an estimate of the difference in the outcome of interest attributable to the intervention. It can be measured in various ways – such as a difference in means or a difference in proportions if the analysis is a before-after analysis or a difference-in-differences for means or proportions if the analysis captures changes over time in the intervention group compared to the comparison group. The difference-in-difference estimates we present are effect sizes.*

Reviewer #2: Thank you for your detailed response to my previous comments. All issues have been addressed satisfactorily.

*Many thanks.*

Reviewer #3: Title: Impact of the Liberian National Community Health Assistant Program on Childhood Illness Care in Grand Bassa County, Liberia: A Difference-in-Differences Analysis of Population-Based Data.

This topic is interesting and the method used is what capture my attention to reading this manuscript. It also shows the pre and post-intervention levels of the national health system of Liberia, showing how they have measured the impact and the consequent focus on the future sustainability of the program. Therefore, this paper is worth publishing.

*Much appreciated.*

The only thing to improve on is for the author to be detailed in the main body of the paper on how they derive the various statistics that were presented in this paper, especially the part of the difference-in-difference analysis.

Thank you.

*Many thanks. We have added some details about how the difference-in-differences were estimated to the text of the methods section. There is more detail on the statistical methods in the methods supplement that deals with the inverse probability of treatment weighting models that we used to adjust the difference-in-differences analysis for potential confounding. (We apologize for not including more on that in the main text of the paper, but it’s fairly lengthy and we suspect it would make the paper more difficult to read for those who are not primarily interested in methods.)*

*We also added some additional text that clarifies how we conducted our sensitivity analysis in which we adjusted the difference-in-difference estimates for confounding via regression adjustment rather than inverse probability of treatment weighting.*
